# Supplementary material for: Bone mineral density and trabecular bone score in elderly type 2 diabetes Southeast Asian patients with severe osteoporotic hip fractures
Source: PLoS One. 2020 Nov 19;15(11):e0241616. doi: 10.1371/journal.pone.0241616 (PMC7676677; doi:10.1371/journal.pone.0241616)
Supplement: S1 Table — (DOCX) [file pone.0241616.s002.docx]

Supplementary Table 1 : Duration( days ) from date of admission to BMD analysis stratified by gender and DM2 status

| **Variable** | **Women (n=518)** | |  | **Men (n=235)** | |  |
| --- | --- | --- | --- | --- | --- | --- |
| **Diabetes Status** | **ND (n=350)** | **DM2 (n=168)** | **p- value** | **ND (n=162)** | **DM2 (n=73)** | **p-value** |
| Duration (days) from date of admission to BMD analysis  Median (25^th^, 75^th^) | 19.50 (5, 191.25) | 13.50 (5, 116.50) | 0.570 | 17 (5, 113) | 19 (4, 105.50) | 0.690 |
