# Supplementary material for: Bone mineral density and trabecular bone score in elderly type 2 diabetes Southeast Asian patients with severe osteoporotic hip fractures
Source: PLoS One. 2020 Nov 19;15(11):e0241616. doi: 10.1371/journal.pone.0241616 (PMC7676677; doi:10.1371/journal.pone.0241616)
Supplement: S2 Table — (DOCX) [file pone.0241616.s003.docx]

Supplementary Table 2 : Correlation between TBS and BMD in DM2 and non DM2 patients stratified by gender

|  | Non-DM2 | | | | | | | | DM2 | | | | | | | |
| --- | --- | --- | --- | --- | --- | --- | --- | --- | --- | --- | --- | --- | --- | --- | --- | --- |
|  | Female | | | | Male | | | | Female | | | | Male | | | |
|  | R | R^2^ | BMI and age-adjusted R | BMI, 25(OH)D, eGFR and age-adjusted R | R | R^2^ | BMI and age-adjusted R | BMI, 25(OH)D, eGFR and age-adjusted R | R | R^2^ | BMI and age-adjusted R | BMI, 25(OH)D, eGFR and age-adjusted R | R | R^2^ | BMI and age-adjusted R | BMI, 25(OH)D, eGFR and age-adjusted R |
| BMD L-spine | 0.358* | 0.128 | 0.413^*^ | 0.416^*^ | 0.511* | 0.261 | 0.585^*^ | 0.591^*^ | 0.415^*^ | 0.173 | 0.467^*^ | 0.439^*^ | 0.459^*^ | 0.211 | 0.523^*^ | 0.511^*^ |
| BMD total Hip | 0.243* | 0.059 | 0.256^*^ | 0.258^*^ | 0.308* | 0.095 | 0.281^*^ | 0.285^*^ | 0.182^*^ | 0.033 | 0.203^*^ | 0.191^*^ | 0.397^*^ | 0.157 | 0.480^*^ | 0.478^*^ |
| BMD F-neck | 0.246* | 0.061 | 0.260^*^ | 0.259^*^ | 0.357* | 0.127 | 0.325^*^ | 0.324^*^ | 0.198^*^ | 0.039 | 0.212^*^ | 0.208^*^ | 0.384^*^ | 0.147 | 0.465^*^ | 0.461^*^ |

^*^ p <0.005
