# Supplementary material for: Bone mineral density and trabecular bone score in elderly type 2 diabetes Southeast Asian patients with severe osteoporotic hip fractures
Source: PLoS One. 2020 Nov 19;15(11):e0241616. doi: 10.1371/journal.pone.0241616 (PMC7676677; doi:10.1371/journal.pone.0241616)
Supplement: S3 Table — (DOCX) [file pone.0241616.s004.docx]

Supplementary Table 3 : Correlation between BMI with TBS and BMD in DM2 and nonDM2 patients stratified by gender

|  | Non-DM2 | | | | DM2 | | | |
| --- | --- | --- | --- | --- | --- | --- | --- | --- |
|  | Female | | Male | | Female | | Male | |
|  | R | R^2^ | R | R^2^ | R | R^2^ | R | R^2^ |
| TBS | -0.027 | 0.001 | -0.005 | 0.00002 | -0.104 | 0.011 | -0.218 | 0.048 |
| BMD L-spine | 0.452^*^ | 0.205 | 0.322^*^ | 0.104 | 0.280^*^ | 0.078 | 0.144 | 0.021 |
| BMD total Hip | 0.434^*^ | 0.188 | 0.375^*^ | 0.141 | 0.274^*^ | 0.075 | 0.255^*^ | 0.065 |
| BMD F-neck | 0.443^*^ | 0.197 | 0.366^*^ | 0.134 | 0.245^*^ | 0.060 | 0.242^*^ | 0.059 |
