# Supplementary material for: Bone mineral density and trabecular bone score in elderly type 2 diabetes Southeast Asian patients with severe osteoporotic hip fractures
Source: PLoS One. 2020 Nov 19;15(11):e0241616. doi: 10.1371/journal.pone.0241616 (PMC7676677; doi:10.1371/journal.pone.0241616)
Supplement: S4 Table — (DOCX) [file pone.0241616.s005.docx]

Supplementary Table 4 : Demographic and Clinical variables of DM2 patients subdivided into gender and DM2 control

|  | Women (n=158) | | | Men (72) | | |
| --- | --- | --- | --- | --- | --- | --- |
|  | < 7% (n=85) | ≥ 7% (n=74) | P value | < 7 % (n=36) | ≥ 7% (n=36) | P value |
| Age (years) | 79.44 + 7.77 | 73.64 + 8.32 | <0.001 | 76.94 + 8.98 | 71.36 + 11.08 | 0.022 |
| Height (cm) | 149.92 + 6.34 | 151.41 + 5.31 | 0.112 | 158.89 + 8.20 | 162.74 + 7.25 | 0.038 |
| Weight (kg) | 52.31 + 10.66 | 52.06 + 10.16 | 0.881 | 55.70 + 10.05 | 59.56 + 12.31 | 0.149 |
| BMI (kg/m^2^) | 23.19 + 4.00 | 22.70 + 4.28 | 0.458 | 22.10 + 3.81 | 22.65 + 5.40 | 0.614 |
| BMI group (Asian)  Underweight (< 18.5)  Normal (18.5-22.9)  Overweight (23-24.9)  Obese (≥ 25) | 7 (8.2)  38 (44.7)  19 (22.4)  21 (24.7) | 11 (14.9)  31 (41.9)  12 (16.2)  20 (27.0) | 0.484 | 5 (13.9)  17 (47.2)  9 (25.0)  5 (25.0) | 8 (22.2)  13 (36.1)  5 (13.9)  10 (27.8) | 0.258 |
| Race  Chinese  Malay  Indian  Others | 60 (70.6)  14 (16.5)  7 (8.2)  4 (4.7) | 42 (56.8)  19 (25.7)  7 (9.5)  6 (8.1) | 0.309 | 23 (63.9)  10 (27.8)  1 (2.8)  2 (5.6) | 23 (63.9)  5 (13.9)  4 (11.1)  4 (11.1) | 0.247 |
| Current smoker | 0 (0.0) | 0 (0.0) | NA | 3 (8.3) | 6 (16.7) | 0.285 |
| Alcohol > 3 units per day | 0 (0.0) | 0 (0.0) | NA | 0 (0.0) | 1 (2.8) | 1.000 |
| Previous fracture | 12 (14.1) | 6 (8.1) | 0.317 | 2 (5.6) | 4 (11.1) | 0.674 |
| Rheumatoid arthritis | 0 (0.0) | 0 (0.0) | NA | 0 (0.0) | 0 (0.0) | NA |
| Secondary osteoporosis | 3 (3.5) | 2 (2.7) | 1.000 | 0 (0.0) | 1 (2.8) | 1.000 |
| Steroids (> 3 months ) | 1 (1.2) | 0 (0.0) | 1.000 | 0 (0.0) | 0 (0.0) | NA |
| Dementia | 14 (16.5) | 10 (13.5) | 0.662 | 5 (13.9) | 4 (11.1) | 1.000 |
| Amputation | 5 (5.9) | 2 (2.7) | 0.451 | 0 (0.0) | 2 (5.6) | 0.493 |
| eGFR | 55.12 + 23.46 | 65.11 + 24.00 | 0.009 | 58.06 + 22.15 | 64.75 + 26.60 | 0.250 |
| CKD (eGFR < 60 ) | 38 (44.7) | 44 (59.5) | 0.063 | 18 (50.0) | 25 (69.4) | 0.093 |
| 25(OH)D (ug/L) | 21.63 + 12.03 | 19.92 + 9.28 | 0.329 | 23.34 + 10.89 | 19.87 + 8.21 | 0.131 |
| Calcium & Vitamin D supplementation | 38 (44.7) | 23 (31.1) | 0.078 | 8 (22.2) | 8 (22.2) | 1.000 |
| HbA1C | 6.06 + 0.65 | 8.29 + 1.68 | <0.001 | 6.18 + 0.54 | 8.62 + 1.64 | <0.001 |
| Length of stay (day) | 10 (7, 16) | 8 (6.75, 12) | 0.020 | 12 (7, 17.75) | 10 (7, 16) | 0.856 |
| Inpatient mortality | 1 (1.2) | 1 (1.4) | 1.000 | 0 (0.0) | 0 (0.0) | NA |
| Metformin | 58 (68.2) | 62 (83.8) | 0.023 | 21 (58.3) | 28 (77.8) | 0.077 |
| Sulphonylurea | 48 (56.5) | 52 (70.3) | 0.072 | 28 (77.8) | 25 (69.4) | 0.422 |
| DPPIV-I | 2 (2.4) | 6 (8.1) | 0.147 | 1 (2.8) | 2 (5.6) | 1.000 |
| Insulin | 5 (5.9) | 13 (17.6) | 0.020 | 1 (2.8) | 9 (25.0) | 0.006 |
| Insulin dose unit per kg | 0.32 + 0.18 | 0.46 + 0.22 | 0.232 | 0.48 (0.0) | 0.31 + 0.17 | 0.380 |
| Neuropathy | 1 (1.2) | 0 (0.0) | 1.000 | 0 (0.0) | 0 (0.0) | NA |
| Microalbuminura | 9 (10.7) | 6 (8.1) | 0.577 | 2 (5.6) | 3 (8.3) | 1.000 |
| Duration of diabetes  Median (IQR) | 2 (1, 3) | 2 (1.75, 3) | 0.314 | 2 (1,3) | 2 (1,3) | 0.481 |
