# Supplementary material for: Bone mineral density and trabecular bone score in elderly type 2 diabetes Southeast Asian patients with severe osteoporotic hip fractures
Source: PLoS One. 2020 Nov 19;15(11):e0241616. doi: 10.1371/journal.pone.0241616 (PMC7676677; doi:10.1371/journal.pone.0241616)
Supplement: S5 Table — a: Relationship of trabecular bone score with diabetes mellitus (DM2) medications, complications and glycaemia control (β Coefficient). b: Relationship of Trabecular Bone Score adjusted for Lumbar Spine BMD with Diabetes Mellitus (DM2) Medication, Complication and Glycaemia Control (β Coefficient). (DOCX) [file pone.0241616.s006.docx]

Supplementary Table 5a: Relationship of trabecular bone score with diabetes mellitus (DM2) medications, complications and glycaemia control (β Coefficient).

.

|  | TBS | | BMD Lumbar Spine | | BMD Total Hip | | BMD Femur Neck | |
| --- | --- | --- | --- | --- | --- | --- | --- | --- |
|  | Women  (n=158) | Men  (n=72) | Women  (n=158) | Men  (n=72) | Women  (n=158) | Men  (n=72) | Women  (n=158) | Men  (n=72) |
| Insulin | 0.08 (0.02, 0.13) ^§^ | 0.004 (-0.09, 0.10) | 0.10 (0.01, 0.18) ^§^ | -0.03 (-0.20, 0.14) | 0.04 (-0.02, 0.10) | -0.05 (-0.18, 0.08) | 0.06 (0.004, 0.11)^§^ | 0.01 (-0.12, 0.14) |
| Metformin | -0.01 (-0.05, 0.04) | -0.03 (-0.08, 0.03) | 0.06 (-0.004, 0.13)^*‡^ | -0.11 (-0.21, 0.01) ^§^ | 0.01 (-0.03, 0.06) | -0.04 (-0.12, 0.04) | 0.03 (-0.01, 0.07) | 0.01 (-0.07, 0.09) |
| Sulphonylurea | 0.03 (-0.01, 0.06) | 0.03 (-0.04, 0.10) | 0.003 (-0.05, 0.06) | 0.05 (-0.07, 0.17) | -0.01(-0.05, 0.03) | 0.03 (-0.06, 0.12) | 0.02 (-0.02, 0.05) | 0.04 (-0.05, 0.12) |
| eGFR < 60 | 0.02 (-0.02, 0.05) | 0.003 (-0.06, 0.06) | -0.03 (-0.08, 0.03) | -0.05 (-0.15, 0.05)^*^ | -0.01 (-0.04, 0.03) | -0.05 (-0.12, 0.03) | 0.01 (-0.03, 0.04) | 0.02 (-0.05, 0.09) |
| Microvascular complication | -0.0003 (-0.04, 0.04) | -0.04 (-0.03, 0.10) | -0.01 (-0.07, 0.05) | 0.05 (-0.06, 0.15) | -0.01 (-0.05, 0.03) | -0.05 (-0.13, 0.04) | -0.03 (-0.07, 0.01) | -0.03 (-0.11, 0.05) |
| Duration of Diabetes | -0.0003 (-0.04, 0.04) | -0.003 (-0.01, 0.01) | 0.0003 (-0.01, 0.01) | 0.01 (-0.01, 0.02) | 0.0004 (-0.01, 0.01) | 0.003 (-0.01, 0.01) | -0.002 (-0.01, 0.003) | -0.001 (-0.01, 0.01) |
| HbA1C ≥ 7 % | -0.04 (-0.08, -0.01) ^§^ | 0.06 (0.001, 0.12)^§^ | -0.04 (-0.10, 0.01) | 0.12 (0.02, 0.22) | 0.01 (-0.03, 0.05) | 0.10 (0.03, 0.18) ^§^ | -0.003 (-0.04, 0.03) | 0.05 (-0.03, 0.12) |

Reference category = No for insulin, metformin, sulphonylurea, eGFR <60 and Microvascular complication, HbA1C <7 %

Multivariate with age, BMI, race, insulin, metformin, sulphonylurea, presence of microvascular complication, duration of diabetes and HbA1c adjusted. *Microvascular complication was defined as patient who had microalbuminuria or amputation or eGFR < 60.*

*p < 0.050 in univariate model.

^‡^p < 0.050 in multivariate model with age and BMI adjusted.

^§^p < 0.050 in multivariate model with age, BMI, race, insulin, metformin, sulphonylurea, presence of microvascular complication, duration of diabetes and HbA1c adjusted

Supplementary Table 5b: Relationship of Trabecular Bone Score adjusted for Lumbar Spine BMD with Diabetes Mellitus (DM2) Medication, Complication and Glycaemia Control (β Coefficient)

|  | TBS | |
| --- | --- | --- |
|  | Women  (n=158) | Men  (n=72) |
| Insulin | 0.05 (-0.003, 0.10) | 0.01 (-0.08, 0.10) |
| Metformin | -0.03 (-0.06, 0.01) | 0.003 (-0.05, 0.06) |
| Sulphonylurea | 0.03 (-0.003, 0.06) | 0.01 (-0.05, 0.08) |
| eGFR < 60 | 0.03 (-0.01, 0.06) | 0.02 (-0.04, 0.07) |
| Microvascular complication | 0.003 (-0.03, 0.04) | 0.03 (-0.03, 0.08) |
| Duration of Diabetes | 0.0002 (-0.004, 0.01) | -0.004 (-0.01, 0.003) |
| HbA1c ≥ 7 | -0.03 (-0.06, 0.004) | 0.03 (-0.03, 0.08) |
| BMD LSpine | 0.32 (0.22, 0.41)^#^ | 0.26 (0.13, 0.40)^#^ |

^#^ p < 0.050 in multivariate model with age, BMI, race, insulin, metformin, sulphonylurea, presence of microvascular complication, duration of diabetes, HbA1c and BMD LS adjusted.
