# Supplementary material for: Bone mineral density and trabecular bone score in elderly type 2 diabetes Southeast Asian patients with severe osteoporotic hip fractures
Source: PLoS One. 2020 Nov 19;15(11):e0241616. doi: 10.1371/journal.pone.0241616 (PMC7676677; doi:10.1371/journal.pone.0241616)
Supplement: S6 Table — (DOCX) [file pone.0241616.s007.docx]

Supplementary table 6 : Interaction term analysis between TBS and DM2 status with gender

|  | TBS | |
| --- | --- | --- |
|  | Mean difference | P value |
| Female * Non-DM2 | -0.14 | <0.001 |
| Female * DM2 | -0.09 | <0.001 |
| Male * Non-DM2 | -0.02 | 0.245 |
| Male * DM2 | REF |  |
