# Supplementary material for: Bone mineral density and trabecular bone score in elderly type 2 diabetes Southeast Asian patients with severe osteoporotic hip fractures
Source: PLoS One. 2020 Nov 19;15(11):e0241616. doi: 10.1371/journal.pone.0241616 (PMC7676677; doi:10.1371/journal.pone.0241616)
Supplement: S7 Table — (DOCX) [file pone.0241616.s008.docx]

Supplementary Table 7: Relationship of trabecular bone score with diabetes mellitus (DM2) medications, complications and glycaemia control (β Coefficient).

|  | TBS | | BMD Lumbar Spine | | BMD Total Hip | | BMD Femur Neck | |
| --- | --- | --- | --- | --- | --- | --- | --- | --- |
|  | Women  (n=158) | Men  (n=72) | Women  (n=158) | Men  (n=72) | Women  (n=158) | Men  (n=72) | Women  (n=158) | Men  (n=72) |
| Insulin | 0.07 (0.10, 0.12) ^§^ | 0.01 (-0.09, 0.11) | 0.10 (0.01, 0.18) ^§^ | 0.05 (-0.13, 0.22) | 0.04 (-0.02, 0.10) | 0.0004 (-0.13, 0.13) | 0.06 (0.01, 0.12)^§^ | 0.002 (-0.13, 0.13) |
| Metformin | -0.01 (-0.05, 0.32) | -0.01 (-0.07, 0.04) | 0.05 (-0.01, 0.11)^*‡^ | -0.07 (-0.17, 0.03) | 0.01 (-0.04, 0.05) | -0.01 (-0.09, 0.06) | 0.03 (-0.01, 0.07) | 0.01(-0.06, 0.08) |
| Sulphonylurea | 0.03 (-0.01, 0.06) | 0.02 (-0.04, 0.09) | 0.01 (-0.05, 0.06) | 0.05 (-0.07, 0.17) | -0.004 (-0.04, 0.04) | 0.03 (-0.06, 0.11) | 0.02 (-0.02, 0.05) | 0.04 (-0.05, 0.12) |
| eGFR < 60 | 0.02 (-0.02, 0.05) | 0.01 (-0.04, 0.07) | -0.03 (-0.08, 0.03) | -0.03 (-0.13, 0.07)^*^ | -0.01 (-0.05, 0.03) | -0.02 (-0.10, 0.06) | -0.01 (-0.04, 0.03) | -0.02 (-0.09, 0.05) |
| Microvascular complication | 0.0002 (-0.04, 0.04) | 0.03 (-0.03, 0.09) | 0.002 (-0.01, 0.06) | 0.05 (-0.06, 0.16) | -0.01 (-0.05, 0.03) | -0.05 (-0.13, 0.03) | -0.03 (-0.07, 0.01) | -0.03 (-0.11, 0.05) |
| Duration of Diabetes | -0.0004 (-0.01, 0.01) | -0.002  (-0.01, 0.01) | -0.0004  (-0.01, 0.01) | 0.003 (-0.01, 0.02) | 0.0001 (-0.01, 0.01) | 0.001 (-0.01, 0.01) | -0.002 (-0.01, 0.003) | -0.001 (-0.01, 0.01) |
| HbA1c | -0.0003 (-0.01, 0.01) | 0.01 (-0.01, 0.03) | -0.002 (-0.02, 0.01) | 0.01 (-0.02, 0.04) | 0.002 (-0.01, 0.01) | 0.01 (-0.01, 0.03) | -0.003 (-0.01, 0.01) | 0.01 (-0.01, 0.04) |
| Log HbA1c | -0.07 (-0.27, 0.13) | 0.19 (-0.13, 0.52) | -0.06 (-0.36, 0.23) | 0.27 (-0.32, 0.85) | 0.07 (-0.14, 0.28) | 0.23 (-0.20, 0.67) | -0.04 (-0.24, 0.15) | 0.26 (-0.16, 0.67) |

Reference category = No for insulin, metformin, sulphonylurea, eGFR <60 and microvascular complication

Multivariate with age, BMI, race, insulin, metformin, sulphonylurea, presence of microvascular complication, duration of diabetes and HbA1c adjusted. *Microvascular complication was defined as patient who had microalbuminuria or amputation or eGFR < 60.*

*p < 0.050 in univariate model.

^‡^p < 0.050 in multivariate model with age and BMI adjusted.

^§^p < 0.050 in multivariate model with age, BMI, race, insulin, metformin, sulphonylurea, presence of microvascular complication, duration of diabetes and HbA1c adjusted.
