# Supplementary material for: Bone mineral density and trabecular bone score in elderly type 2 diabetes Southeast Asian patients with severe osteoporotic hip fractures
Source: PLoS One. 2020 Nov 19;15(11):e0241616. doi: 10.1371/journal.pone.0241616 (PMC7676677; doi:10.1371/journal.pone.0241616)
Supplement: Attachment — Submitted filename: Editorreply170820.docx [file pone.0241616.s010.docx]

Dear Plos One Editor

We thank you for the opportunity to revise our manuscript and detail here our replies to the reviewers. We thank our reviewers for their time in reviewing this revision and hope this would be sufficient to answer their queries .

Reviewer #3: The paper has been significantly improved.
Please add on table 2 the age and BMI for each categories.

Thank you for the comment , we have added age and BMI into table 2

It can be misleading sometime in the text… when you are saying “elderly patients with DM2 and severe OP present with Hip fracture…. Compared to non DM2 patients”. It could almost applied that the non DM2 patients are not OP with hip fracture. So please throughout the manuscript, abstract and table make it clear….In patients with severe osteoporosis present with Hip fracture: DM2 vs non DM2

Thank you for the comment , we have amended these accordingly to make it clear that these are DM2 and non DM2 patients with hip fracture .

The fact that in poorly controlled women the TBS is lower than well controlled is now consistent with literature…. and clearly have clinical implication. This findings is already important and could be also highlighted in the abstract. Maybe by linking that with the results in men dilute the message. You may want to separate the sentence in two.

Thank you for the comment, we have amended this in the abstract and hope that this clarifies the point better

The fact that HbA1C as continuous variable does not work as good as category could be related to the non-gaussian distribution of the parameter (skewed)…. The log should then be used… (possible explanation….)

Thank you for the comment and suggestion, we re-analyzed the multivariable regression using a log-transformation of the HbA1C and found no association of Log HbA1C with TBS. This is included in supplementary table 7. We have included this explanation in the body of the text to clarify this point.

Yours Sincerely

Corresponding author

Linsey Gani
